# Fulminant Takotsubo syndrome with cardiogenic shock and mitral-edge-to-edge repair in new severe secondary mitral valve regurgitation after elective implantation of a cardiac resynchronization therapy: a case report

**DOI:** 10.1093/ehjcr/ytaf323

**Published:** 2025-07-14

**Authors:** Philipp Steinhoff, Roland Schmitz, Christian Burger, Dirk Westermann, Thomas Arentz

**Affiliations:** Department of Cardiology and Angiology, Faculty of Medicine, Medical Center-University of Freiburg, University of Freiburg, Südring 15, Bad Krozingen 79189, Germany; Department of Cardiology and Angiology, Faculty of Medicine, Medical Center-University of Freiburg, University of Freiburg, Südring 15, Bad Krozingen 79189, Germany; Department of Cardiology and Angiology, Faculty of Medicine, Medical Center-University of Freiburg, University of Freiburg, Südring 15, Bad Krozingen 79189, Germany; Department of Cardiology and Angiology, Faculty of Medicine, Medical Center-University of Freiburg, University of Freiburg, Südring 15, Bad Krozingen 79189, Germany; Department of Cardiology and Angiology, Faculty of Medicine, Medical Center-University of Freiburg, University of Freiburg, Südring 15, Bad Krozingen 79189, Germany

**Keywords:** Takotsubo syndrome, Cardiogenic shock, Mitral regurgitation, Mitral edge-to-edge repair, Case report

## Abstract

**Background:**

Takotsubo syndrome is a cardiac disease typically characterized by transient ventricular dysfunction. Although long considered benign due to the often reversible course, severe cases, including cardiogenic shock, may occur partially due to cardiac complications, such as severe secondary mitral regurgitation.

**Case summary:**

An 81-year-old female patient presented for the elective implantation of cardiac resynchronization therapy (CRT) in preparation of a His-ablation for therapy-refractory, highly symptomatic, paroxysmal atrial fibrillation. Postoperatively, she developed increasing haemodynamic instability, progressing to cardiogenic shock. Echocardiography ruled out pericardial effusion but showed newly developed, significantly reduced left ventricular function as well as new severe secondary mitral regurgitation. Relevant coronary stenosis was ruled out by coronary angiography. Invasive levocardiography revealed the typical pattern of Takotsubo syndrome. Haemodynamic stabilization was achieved by mechanical circulatory support, using the Impella CP device. Attempts to gradually reduce the support level repeatedly failed due to worsening of the mitral regurgitation resulting in pulmonary oedema. Therefore, with no signs for recovery of left ventricular function at that time and considering the patient’s severe symptom burden, an urgent mitral edge-to-edge repair was performed with successful reduction of mitral regurgitation. The haemodynamic situation subsequently stabilized, allowing for the successful weaning and eventually removal of the temporary circulatory support after 5 days. Left ventricular function eventually showed near-complete recovery after 15 days.

**Discussion:**

Even after elective cardiac procedures, such as CRT implantation, fulminant Takotsubo syndrome should be considered as a differential diagnosis in cases of haemodynamic instability. Then, temporary mechanical circulatory support and interventional treatment of secondary complications, such as secondary mitral regurgitation, may be necessary for stabilization.

Learning points

- Takotsubo cardiomyopathy should be considered in the differential diagnosis of haemodynamic instability up to cardiogenic shock even following elective cardiac procedures, such as pacemaker or cardiac resynchronization therapy implantation.
- Early indication for interventional treatment—such as edge-to-edge repair in new-onset mitral regurgitation—should be assessed in case of secondary complications hindering haemodynamic stabilization.

## Introduction

Takotsubo cardiomyopathy or -syndrome (TTS) is a cardiac condition characterized by typically transient abnormal wall motion, most often affecting the apical left ventricular region.^1^ The condition is generally associated with psychological or physical stressors,^2^ with a higher incidence among postmenopausal women.^3^ In recent years, however, it has also been linked to other triggers, such as pacemaker implantations.^4–6^ Despite the usually reversible character more severe courses may occur, including cardiogenic shock.^2,7,8^ These outcomes may result from various secondary complications, such as severely impaired left ventricular function or acute secondary mitral regurgitation.^4,8,9^ The treatment approach is generally supportive.

## Summary figure

**Figure:**
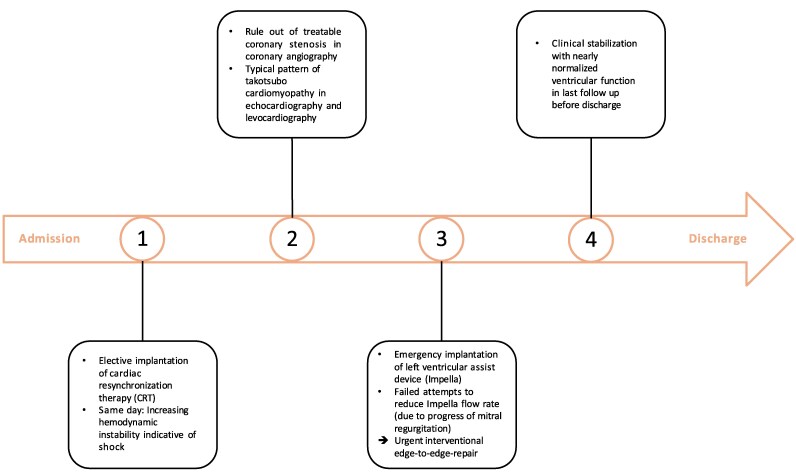


## Case presentation

An 81-year-old, yet age-appropriate, and active patient presented electively in December 2024 for the implantation of cardiac resynchronization therapy (CRT) in preparation for a His-ablation due to therapy-refractory, paroxysmal, highly symptomatic atrial fibrillation. This had been treated with pulmonary vein isolation in 2009, 2012, and 2013, by ablation of other left atrial foci in 2016 and numerous electrical cardioversions. Several attempts with different antiarrhythmic medications had to be abandoned due to ineffectiveness or adverse effects. Upon admission, the patient showed sinus rhythm in ECG and stated current well-being.

The only other significant pre-existing cardiac condition was a severe tricuspid valve regurgitation, which had so far been treated conservatively, as well as a moderate secondary mitral valve regurgitation (vena contracta at admission 4–5 mm) (see Supplementary material online, *Video S1*) due to annular dilatation (38 × 36 mm), both of which were repeatedly stable for many years. Echocardiography upon admission further showed good biventricular function (left ventricular ejection fraction visually estimated > 50%, fractional shortening 40%, TAPSE 26 mm) with significantly increased left atrial volume (93 mL, volume index 49 mL/m²) and normal right ventricular size (basal diameter of 40 mm).

The CRT implantation was successfully performed without initially apparent complications. However, a few hours later, the patient became increasingly haemodynamically unstable. Auscultation revealed bilateral moist rales, indicating pulmonary oedema. Despite initial treatment, symptoms progressed, with an increase in heart rate to 110/min and a drop in blood pressure to 60/40 mmHg, indicative of shock. A venous blood gas analysis showed elevated lactate (3.5 mmol/L). Neurologically, the patient only had a single brief syncope. Mechanical ventilation was not required.

Emergency echocardiography ruled out pericardial effusion but revealed a severely reduced left ventricular function (visually estimated at 30%–35%) due to akinesia of the apical two-thirds, accompanied by a fulminant secondary mitral regurgitation [biplane vena contracta 10 mm (16 × 4 mm)] with a lack of leaflet coaptation and a visible gap caused by chordae traction (see Supplementary material online, *Video S2*). Right ventricular function remained normal. There was no left ventricular outflow tract obstruction or systolic anterior motion of the mitral valve. Laboratory analysis showed significantly increased cardiac ischaemia markers (troponin 509 ng/L from 10.8 ng/L, creatine kinase 400 U/L from 62 U/L, creatine kinase-MB 39 U/L from 10 U/L), prompting an emergency coronary angiography which excluded intervention-requiring coronary stenosis. Levocardiography confirmed the characteristic contraction pattern of TTS.

During deteriorating cardiogenic shock (SKAI D) with refractory hypotension, temporary circulatory support (Impella CP) was established. Repeated attempts in the following 4 days to reduce the support level failed, even with concomitant levosimendan therapy, mainly due to significant worsening of the mitral regurgitation with subsequent pulmonary oedema. Despite the mostly reversible nature of TTS, there were no echocardiographic indications of functional recovery at that time. Given the fulminant clinical course and the patient’s severe symptomatic distress, a continued conservative approach was deemed unreasonable. Consequently, an urgent intervention using edge-to-edge repair was performed (see Supplementary material online, *Video S4*), significantly reducing mitral regurgitation, as evidenced by a decreased left atrial pressure (29/35/23–20/25/17 mmHg). In the pre-interventional transoesophageal echocardiography, the length of the posterior mitral leaflet was 12–13 mm (see Supplementary material online, *Video S3*). Haemodynamic stabilization was swift afterwards. The mechanical circulatory support was withdrawn successfully 5 days after implantation. Post-interventionally, the transmitral gradient was measured at 2 mmHg, indicating no signs of relevant mitral stenosis.

Final echocardiography 15 days after onset of TTS then showed a largely recovered left ventricular function and significantly reduced mitral regurgitation (see Supplementary material online, *Video S5*). The patient was discharged in stable condition and referred for follow-up rehabilitation therapy. When contacted 3 months later, the patient stated well-being and expressed deep gratitude.

## Discussion

This case highlights that even after elective cardiac procedures, like pacemaker implantations, fulminant forms of TTS may occur. Affected individuals might benefit from prompt interventional treatment of secondary complications, such as valve regurgitation.

Strangio *et al*. conducted a systematic review of the occurrence of TTS following pacemaker implantation. Altogether, only 28 patients (average age 74 years, 75% female) were reported, indicating the rare association. One single-centre registry reported TTS in 9 out of 1655 patients (0.54%) after pacemaker implantation.^5^ In Strangio *et al*.’s review, complete recovery of cardiac function was reported in 92%. Haemodynamic instability occurred in 4 out of 28 patients (14%). The overall mortality rate was 3.6%.^6^ It is important to note, however, that all patients underwent pacemaker implantation for an acute rhythmological indication (68% high-grade AV block or atrial fibrillation with slow ventricular response, 32% sick sinus syndrome). In contrast, pacemaker implantation in our patient was performed electively for therapy-resistant atrial fibrillation. Upon admission, the patient was even in sinus rhythm and asymptomatic.

Altogether, the current literature states that severe clinical courses in TTS, including cardiogenic shock, are more frequent than assumed with an incidence of ∼10%.^11^ Some studies even report comparable rates of cardiogenic shock and mortality to those in patients with acute coronary syndrome receiving guideline-compliant treatment.^7^

Another review addresses the occurrence and management of cardiogenic shock in TTS.^8^ Due to the usually spontaneous recovery of cardiac function, a primarily conservative approach is recommended. Various mechanisms can contribute to cardiogenic shock in TTS, including left ventricular failure and acute mitral regurgitation. In our patient, these were the leading mechanisms. Significant mitral regurgitation is reported in 15%–25% of TTS cases.^9^ Urgent interventional treatment has not been described in this context and is therefore not yet part of recommendations. However, considering the clinical course in our patient, such an approach could be crucial.

Regardless the cause, the implantation of an Impella CP should be considered.^8^ In a retrospective registry, 81% of the included 16 patients with cardiogenic shock due to TTS survived under Impella therapy.^10^ Regarding pharmacological options, catecholamines and especially inotropes are usually administered in acute heart failure.^12^ However, in cases due to TTS, this strategy is not recommended, as a possible aetiological connection with catecholamine-induced myocardial stunning and inflammation has been discussed.^12,13^ Therefore, non-catecholaminergic inotropes, such as levosimendan, should be preferred.^10^

## Conclusion

Even following elective cardiac procedures, such as pacemaker implantation, fulminant Takotsubo cardiomyopathy should be considered in the differential diagnosis of haemodynamic instability up to cardiogenic shock. Possible contributing mechanisms include an acute impairment of left ventricular function and secondary mitral regurgitation. For stabilization, the early use of a temporary circulatory support should be considered. Pharmacologically, non-catecholaminergic inotropes should be primarily used. In case of secondary complications, such as new-onset mitral regurgitation hindering stabilization, an early indication for interventional treatment—such as edge-to-edge repair—should be assessed.

## Supplementary Material

ytaf323_Supplementary_Data

## Data Availability

All data are incorporated into the article and its online Supplementary material. The patient’s completed consent form will be retained by the treating facility in accordance with locally approved procedures and stored with the patient’s medical records.

## Lead author biography

After completing my medical studies, I began my professional training in cardiology at the University Heart Center Freiburg-Bad Krozingen in early 2024. During my studies, I developed a strong interest in cardiovascular medicine, which ultimately led me to pursue a career in this field. In addition to my academic education, I was actively involved in research and successfully obtained my doctoral degree shortly after graduating. I am passionate about advancing my expertise in cardiology and contributing to innovative developments in patient care and cardiovascular research.

## Supplementary material

**Consent:** Written informed consent was obtained from the patient for publication of this case report in accordance with the COPE guidelines and is available.

**Funding:** There was no funding involved in this case report.
